# Obituary

**Published:** 1852-12

**Authors:** 


### OBITUARY.

DR. DANIEL DRAKE, the distinguished and venerable author and teacher, whose biography would be a history of the rise and progress of medical science in the West, died at his residence in Cincinatti during the last month.

DR. JONATHAN COWDERY, the oldest surgeon in the U. S. Navy, died recently at Norfolk, aged 85 years.

